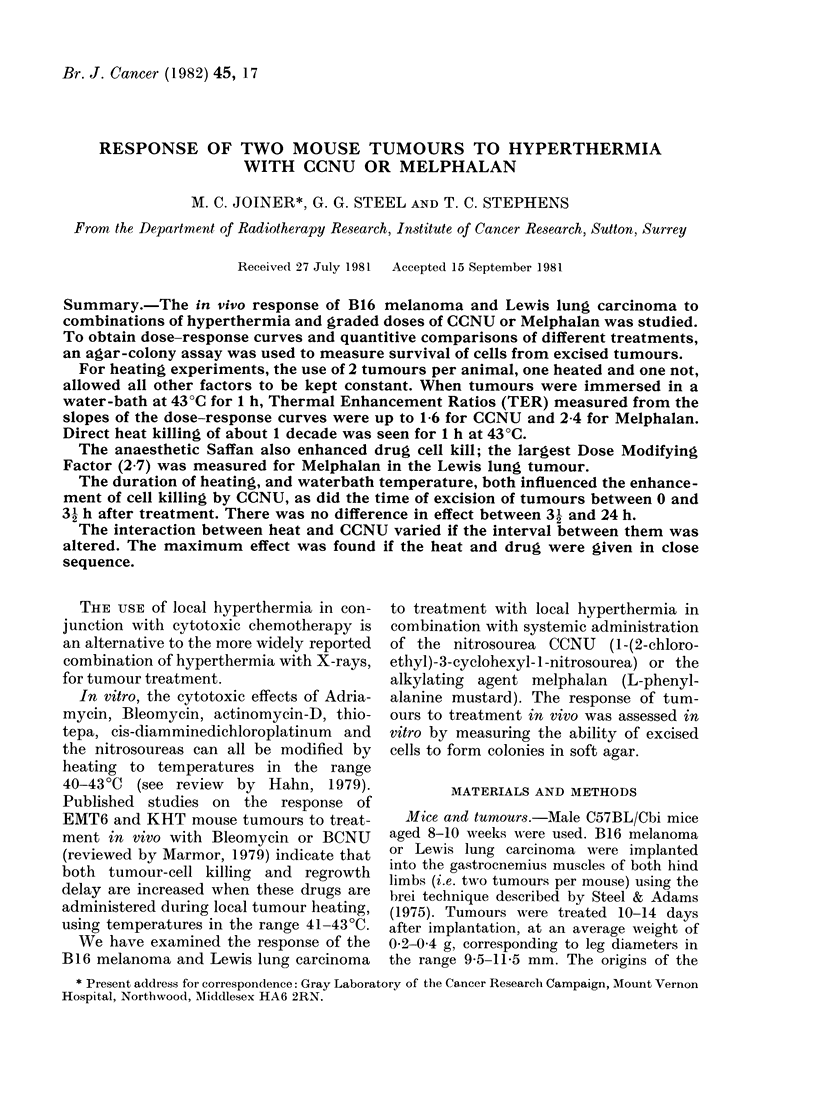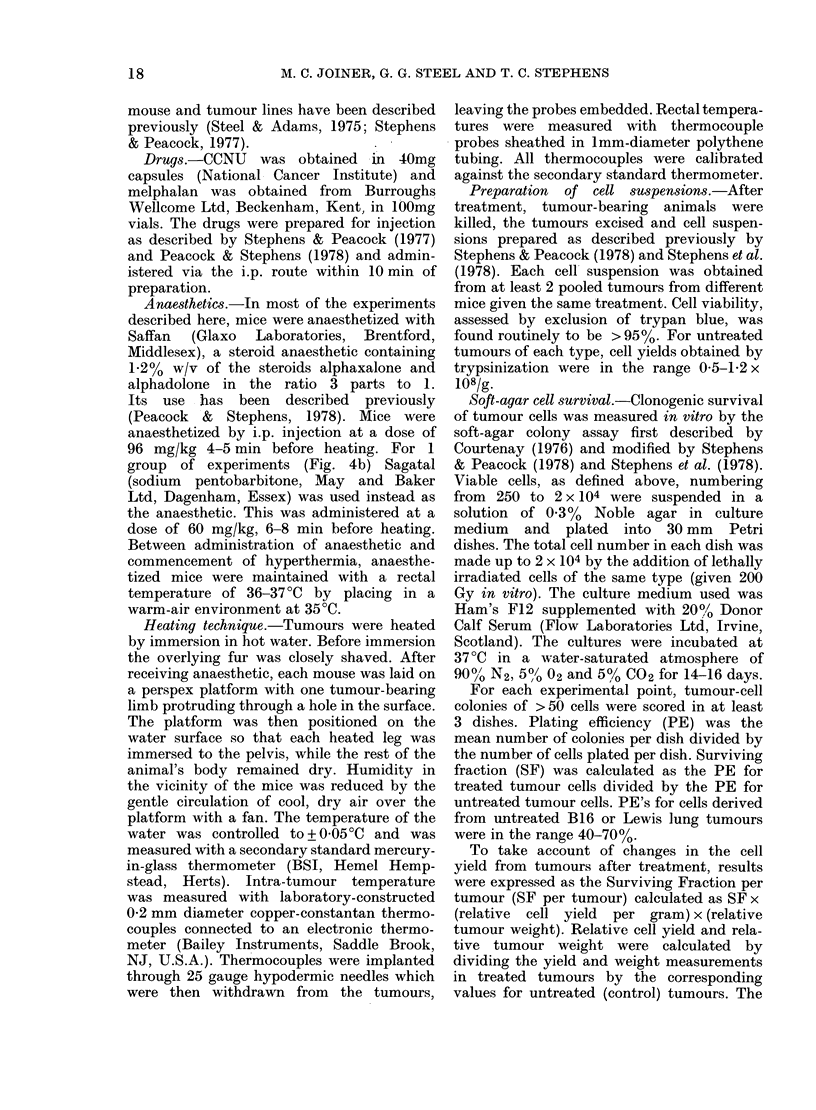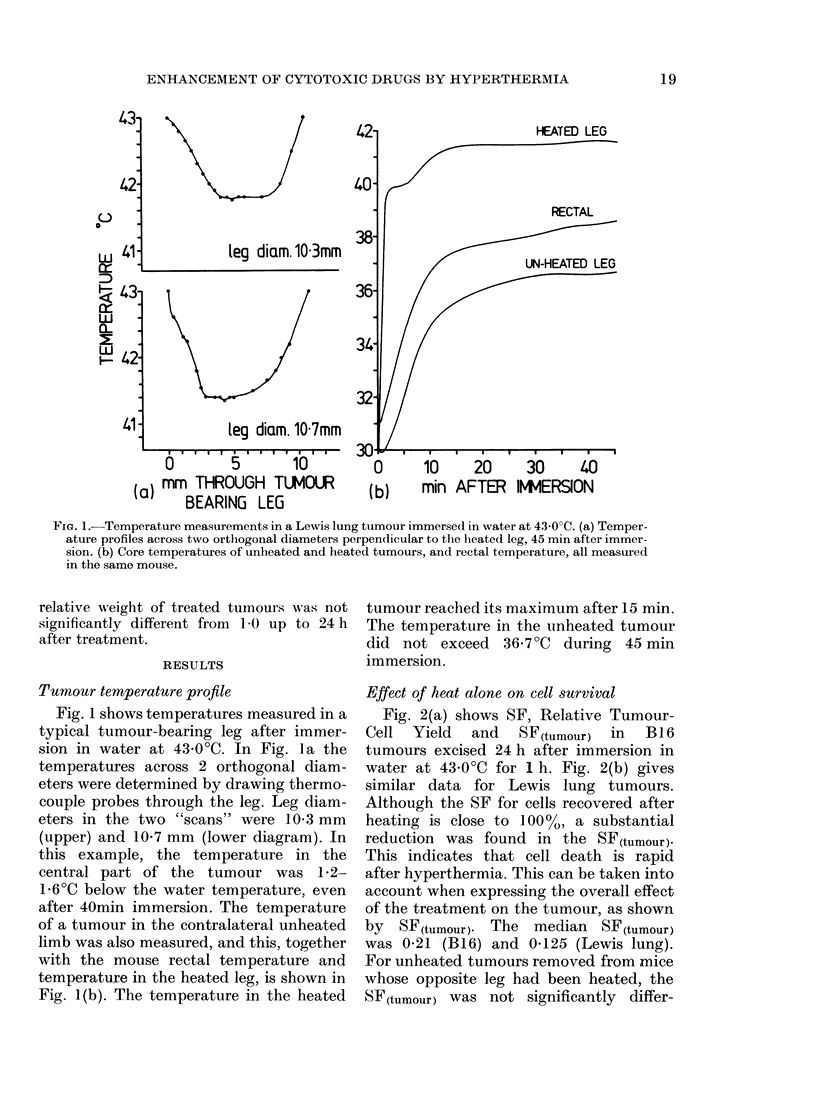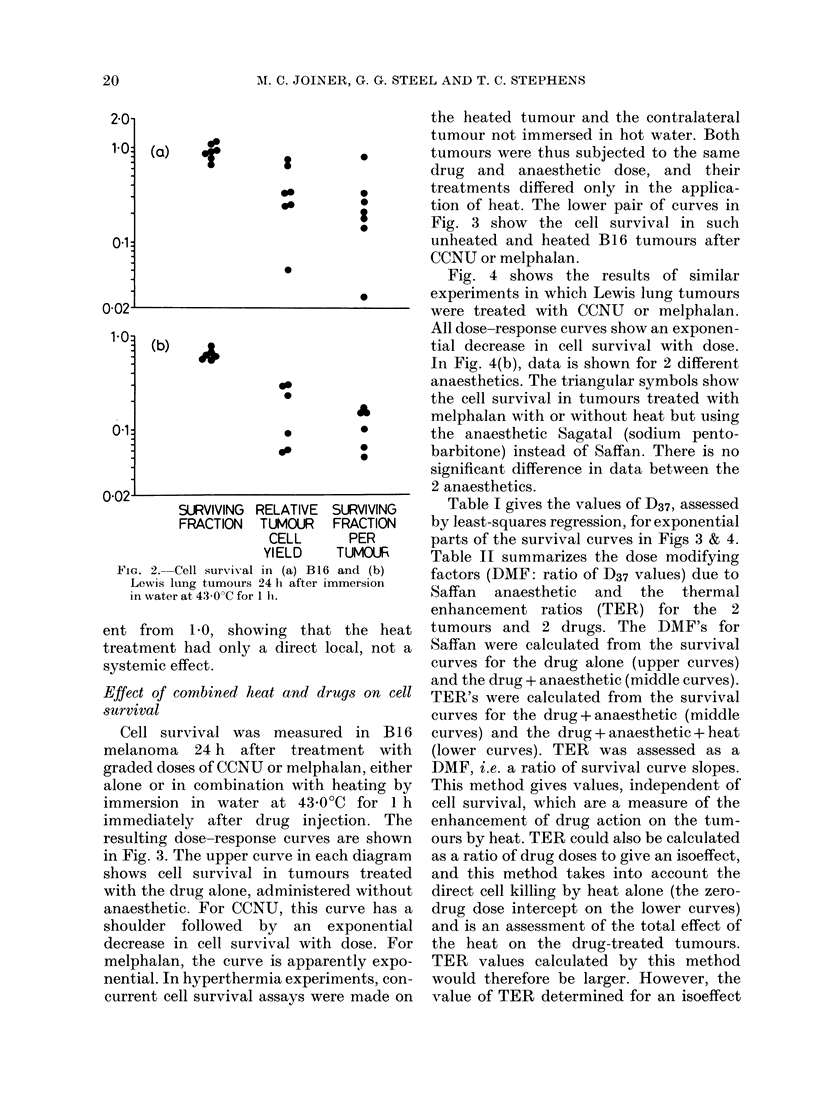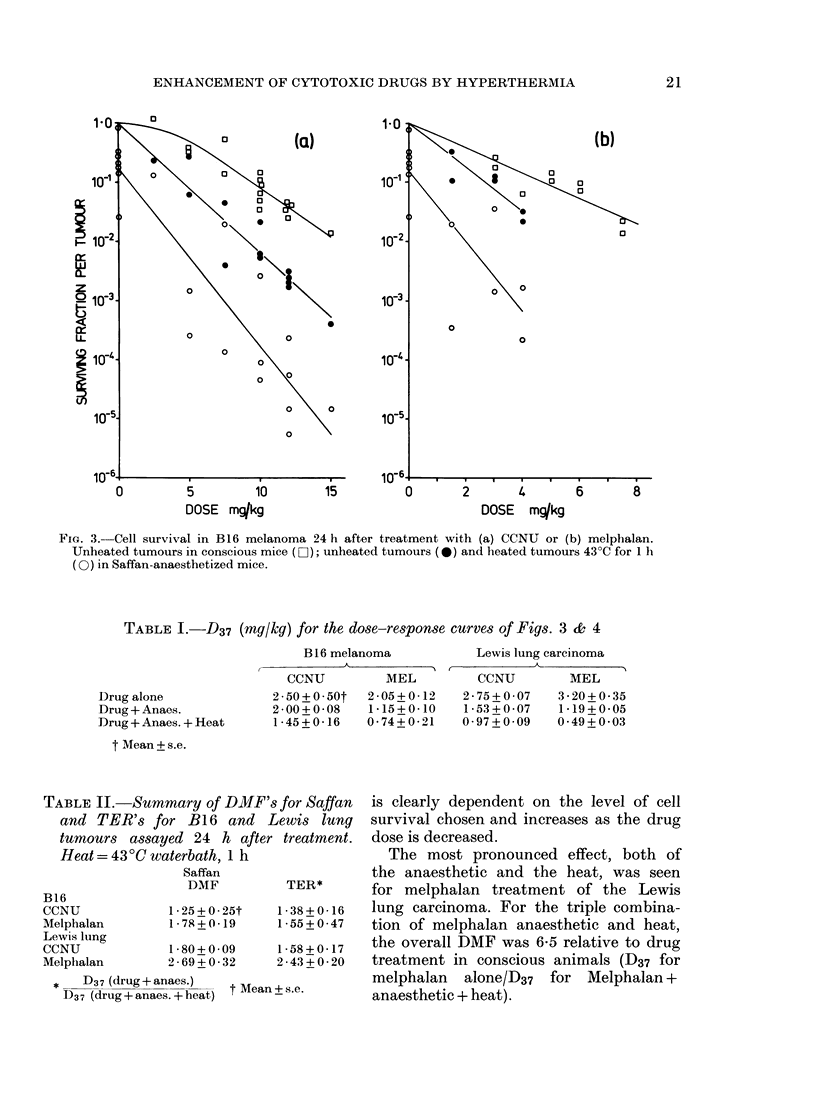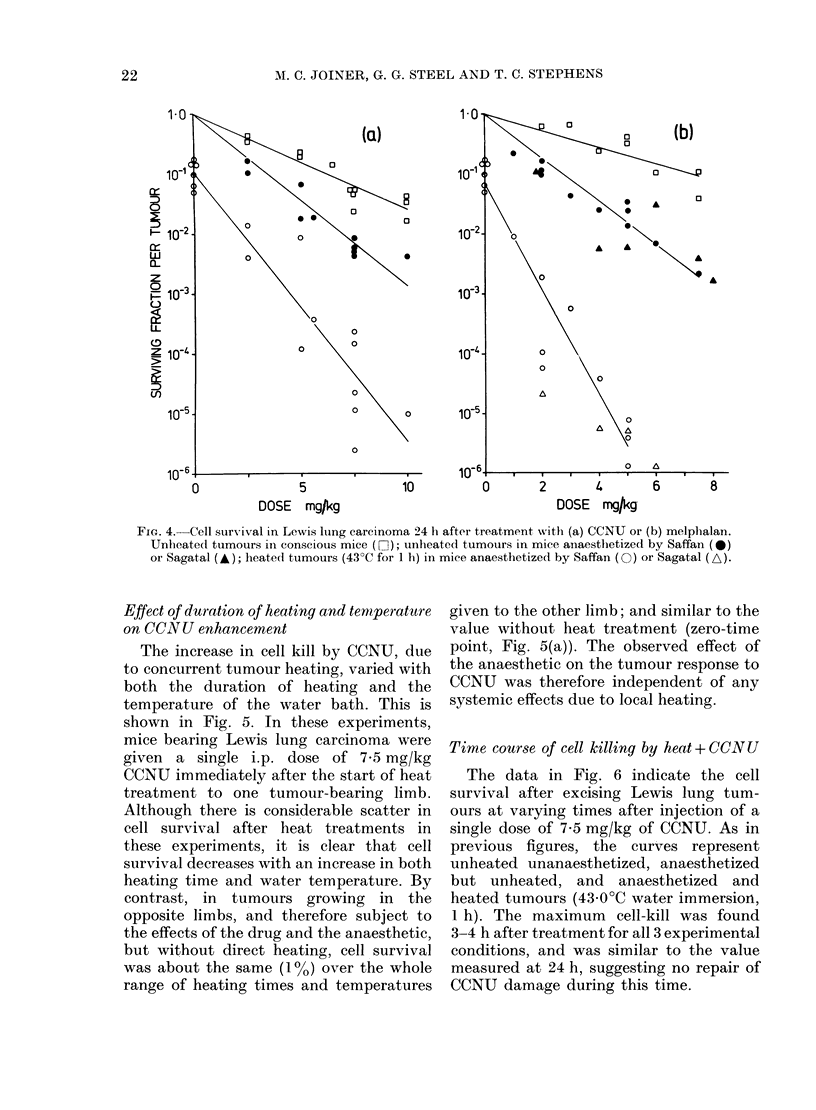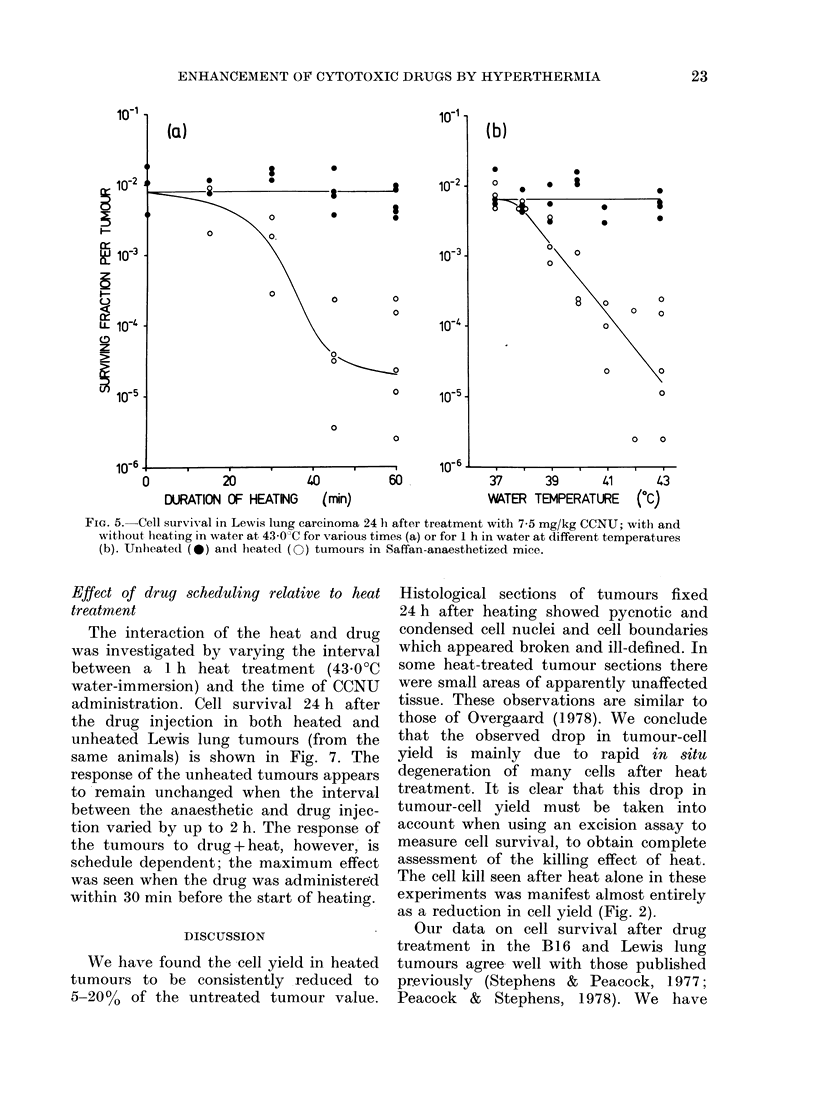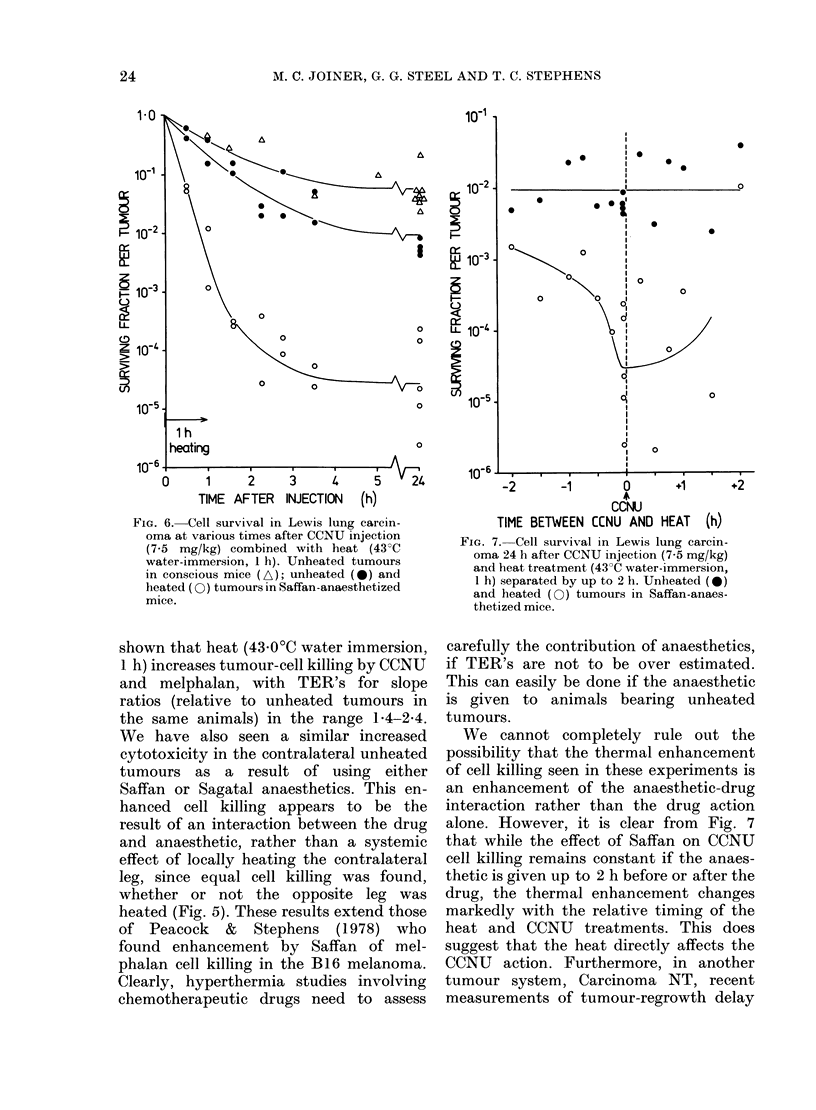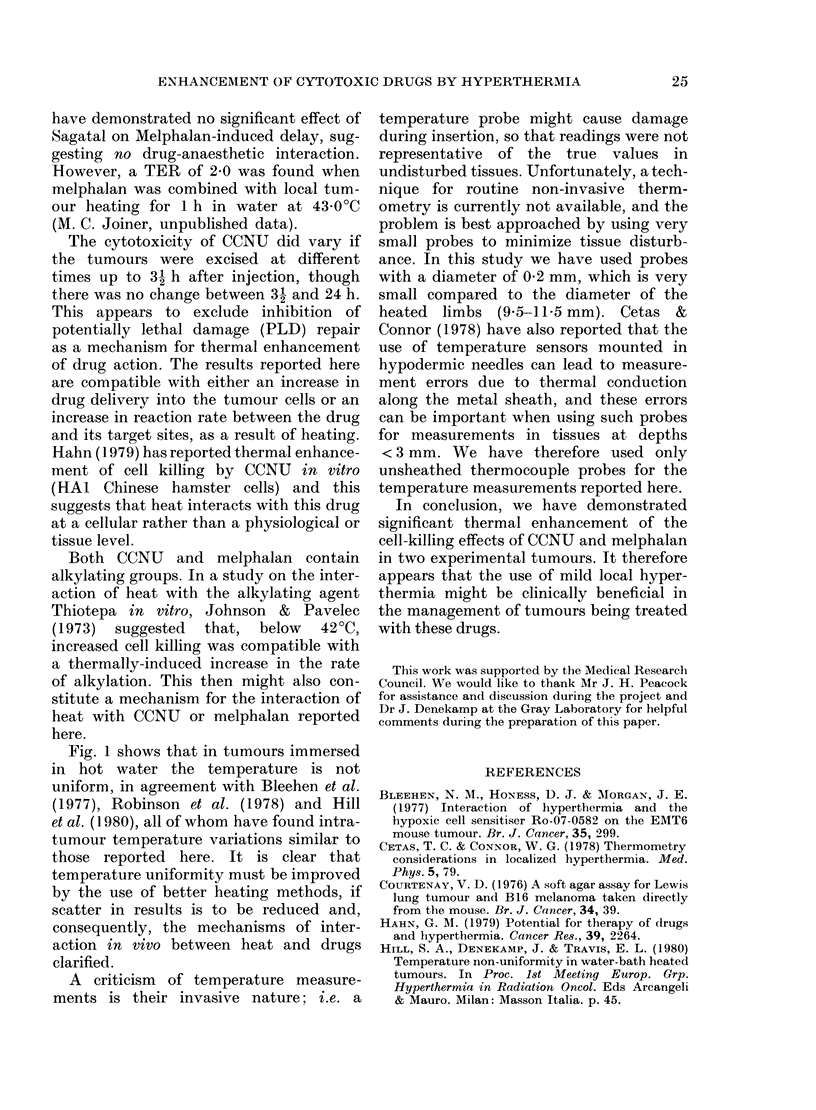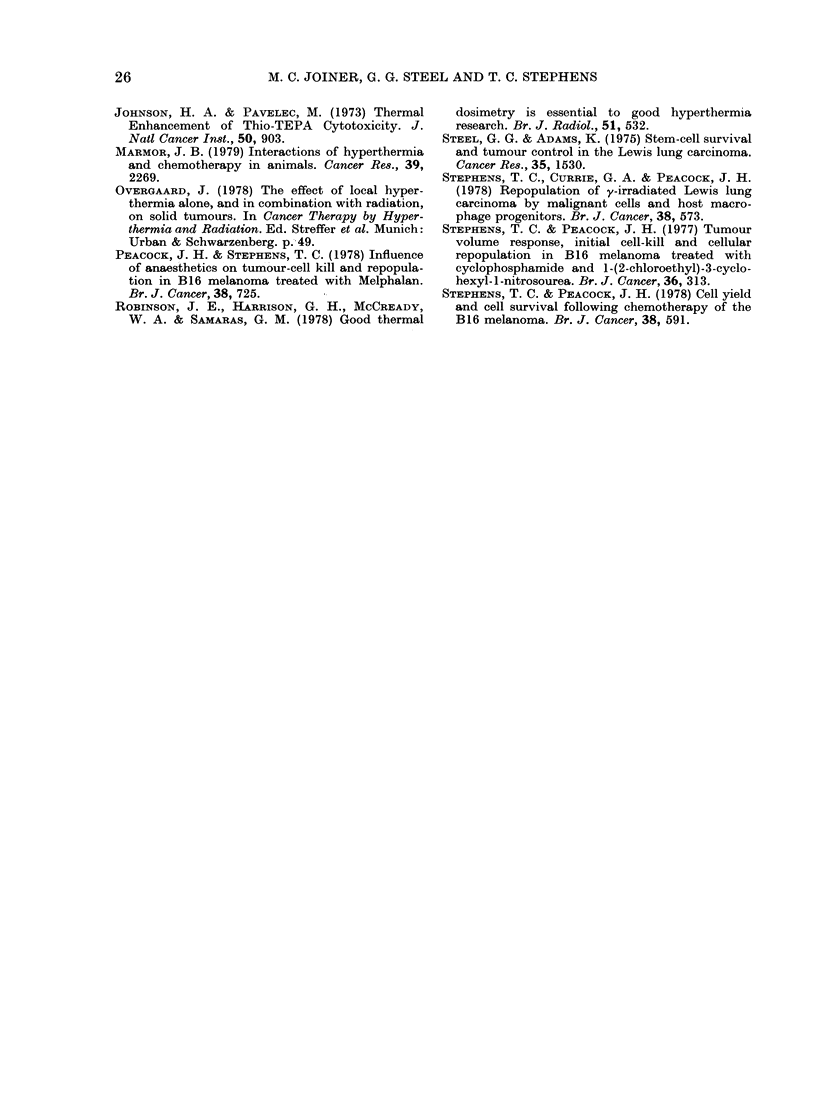# Response of two mouse tumours to hyperthermia with CCNU or melphalan.

**DOI:** 10.1038/bjc.1982.3

**Published:** 1982-01

**Authors:** M. C. Joiner, G. G. Steel, T. C. Stephens

## Abstract

The in vivo response of B16 melanoma and Lewis lung carcinoma to combinations of hyperthermia and graded doses of CCNU or Melphalan was studied. To obtain dose-response curves and quantitative comparisons of different treatments, an agar-colony assay was used to measure survival of cells from excised tumours. For heating experiments, the use of 2 tumours per animal, one heated and one not, allowed all other factors to be kept constant. When tumours were immersed in a water-bath at 43 degrees C for 1 h, Thermal Enhancement Ratios (TER) measured from the slopes of the dose-response curves were up to 1.6 for CCNU and 2.4 for Melphalan. Direct heat killing of about 1 decade was seen for 1 h at 43 degrees C. The anaesthetic Saffan also enhanced drug cell kill; the largest Dose Modifying Factor (2.7) was measured for Melphalan in the Lewis lung tumour. The duration of heating, and waterbath temperature, both influenced the enhancement of cell killing by CCNU, as did the time of excision of tumours between 0 and 3 1/2 h after treatment. There was no difference in effect between 3 1/2 and 24 h. The interaction between heat and CCNU varied if the interval between them was altered. The maximum effect was found if the heat and drug were given in close sequence.


*Br. J. Cancer* (1982) **45**, 17

### RESPONSE OF TWO MOUSE TUMOURS TO HYPERTHERMIA WITH CCNU OR MELPHALAN

M. C. JOINER*, G. G. STEEL AND T. C. STEPHENS

*From the Department of Radiotherapy Research, Institute of Cancer Research, Sutton, Surrey*

Received 27 July 1981 Accepted 15 September 1981

**Summary.—The *in vivo* response of B16 melanoma and Lewis lung carcinoma to combinations of hyperthermia and graded doses of CCNU or Melphalan was studied. To obtain dose–response curves and quantitive comparisons of different treatments, an agar-colony assay was used to measure survival of cells from excised tumours.**

**For heating experiments, the use of 2 tumours per animal, one heated and one not, allowed all other factors to be kept constant. When tumours were immersed in a water-bath at 43°C for 1 h, Thermal Enhancement Ratios (TER) measured from the slopes of the dose–response curves were up to 1·6 for CCNU and 2·4 for Melphalan. Direct heat killing of about 1 decade was seen for 1 h at 43°C.**

**The anaesthetic Saffan also enhanced drug cell kill; the largest Dose Modifying Factor (2·7) was measured for Melphalan in the Lewis lung tumour.**

**The duration of heating, and waterbath temperature, both influenced the enhancement of cell killing by CCNU, as did the time of excision of tumours between 0 and 3½ h after treatment. There was no difference in effect between 3½ and 24 h.**

**The interaction between heat and CCNU varied if the interval between them was altered. The maximum effect was found if the heat and drug were given in close sequence.**

THE USE of local hyperthermia in conjunction with cytotoxic chemotherapy is an alternative to the more widely reported combination of hyperthermia with X-rays, for tumour treatment.

*In vitro,* the cytotoxic effects of Adriamycin, Bleomycin, actinomycin-D, thiotepa, cis-diamminedichloroplatinum and the nitrosoureas can all be modified by heating to temperatures in the range 40–43°C (see review by Hahn, 1979). Published studies on the response of EMT6 and KHT mouse tumours to treatment *in vivo* with Bleomycin or BCNU (reviewed by Marmor, 1979) indicate that both tumour-cell killing and regrowth delay are increased when these drugs are administered during local tumour heating, using temperatures in the range 41–43°C.

We have examined the response of the B16 melanoma and Lewis lung carcinoma to treatment with local hyperthermia in combination with systemic administration of the nitrosourea CCNU (1-(2-chloroethyl)-3-cyclohexyl-1-nitrosourea) or the alkylating agent melphalan (L-phenylalanine mustard). The response of tumours to treatment *in vivo* was assessed *in vitro* by measuring the ability of excised cells to form colonies in soft agar.

### MATERIALS AND METHODS

*Mice and tumours.*—Male C57BL/Cbi mice aged 8–10 weeks were used. B16 melanoma or Lewis lung carcinoma were implanted into the gastrocnemius muscles of both hind limbs (*i.e.* two tumours per mouse) using the brei technique described by Steel & Adams (1975). Tumours were treated 10–14 days after implantation, at an average weight of 0·2–0·4 g, corresponding to leg diameters in the range 9·5–11·5 mm. The origins of the

* Present address for correspondence: Gray Laboratory of the Cancer Research Campaign, Mount Vernon Hospital, Northwood, Middlesex HA6 2RN.

mouse and tumour lines have been described previously (Steel & Adams, 1975; Stephens & Peacock, 1977).

*Drugs.*—CCNU was obtained in 40mg capsules (National Cancer Institute) and melphalan was obtained from Burroughs Wellcome Ltd, Beckenham, Kent, in 100mg vials. The drugs were prepared for injection as described by Stephens & Peacock (1977) and Peacock & Stephens (1978) and administered via the i.p. route within 10 min of preparation.

*Anaesthetics.*—In most of the experiments described here, mice were anaesthetized with Saffan (Glaxo Laboratories, Brentford, Middlesex), a steroid anaesthetic containing 1·2% w/v of the steroids alphaxalone and alphadolone in the ratio 3 parts to 1. Its use has been described previously (Peacock & Stephens, 1978). Mice were anaesthetized by i.p. injection at a dose of 96 mg/kg 4–5 min before heating. For 1 group of experiments (Fig. 4b) Sagatal (sodium pentobarbitone, May and Baker Ltd, Dagenham, Essex) was used instead as the anaesthetic. This was administered at a dose of 60 mg/kg, 6–8 min before heating. Between administration of anaesthetic and commencement of hyperthermia, anaesthetized mice were maintained with a rectal temperature of 36–37°C by placing in a warm-air environment at 35°C.

*Heating technique.*—Tumours were heated by immersion in hot water. Before immersion the overlying fur was closely shaved. After receiving anaesthetic, each mouse was laid on a perspex platform with one tumour-bearing limb protruding through a hole in the surface. The platform was then positioned on the water surface so that each heated leg was immersed to the pelvis, while the rest of the animal's body remained dry. Humidity in the vicinity of the mice was reduced by the gentle circulation of cool, dry air over the platform with a fan. The temperature of the water was controlled to $\pm 0{\cdot}05$°C and was measured with a secondary standard mercury-in-glass thermometer (BSI, Hemel Hempstead, Herts). Intra-tumour temperature was measured with laboratory-constructed 0·2 mm diameter copper-constantan thermocouples connected to an electronic thermometer (Bailey Instruments, Saddle Brook, NJ, U.S.A.). Thermocouples were implanted through 25 gauge hypodermic needles which were then withdrawn from the tumours, leaving the probes embedded. Rectal temperatures were measured with thermocouple probes sheathed in 1mm-diameter polythene tubing. All thermocouples were calibrated against the secondary standard thermometer.

*Preparation of cell suspensions.*—After treatment, tumour-bearing animals were killed, the tumours excised and cell suspensions prepared as described previously by Stephens & Peacock (1978) and Stephens *et al.* (1978). Each cell suspension was obtained from at least 2 pooled tumours from different mice given the same treatment. Cell viability, assessed by exclusion of trypan blue, was found routinely to be $>95\%$. For untreated tumours of each type, cell yields obtained by trypsinization were in the range $0{\cdot}5$–$1{\cdot}2 \times 10^8$/g.

*Soft-agar cell survival.*—Clonogenic survival of tumour cells was measured *in vitro* by the soft-agar colony assay first described by Courtenay (1976) and modified by Stephens & Peacock (1978) and Stephens *et al.* (1978). Viable cells, as defined above, numbering from 250 to $2 \times 10^4$ were suspended in a solution of 0·3% Noble agar in culture medium and plated into 30 mm Petri dishes. The total cell number in each dish was made up to $2 \times 10^4$ by the addition of lethally irradiated cells of the same type (given 200 Gy *in vitro*). The culture medium used was Ham's F12 supplemented with 20% Donor Calf Serum (Flow Laboratories Ltd, Irvine, Scotland). The cultures were incubated at 37°C in a water-saturated atmosphere of 90% $N_2$, 5% $O_2$ and 5% $CO_2$ for 14–16 days.

For each experimental point, tumour-cell colonies of $>50$ cells were scored in at least 3 dishes. Plating efficiency (PE) was the mean number of colonies per dish divided by the number of cells plated per dish. Surviving fraction (SF) was calculated as the PE for treated tumour cells divided by the PE for untreated tumour cells. PE's for cells derived from untreated B16 or Lewis lung tumours were in the range 40–70%.

To take account of changes in the cell yield from tumours after treatment, results were expressed as the Surviving Fraction per tumour (SF per tumour) calculated as SF $\times$ (relative cell yield per gram) $\times$ (relative tumour weight). Relative cell yield and relative tumour weight were calculated by dividing the yield and weight measurements in treated tumours by the corresponding values for untreated (control) tumours. The

FIG. 1.—Temperature measurements in a Lewis lung tumour immersed in water at 43·0°C. (a) Temperature profiles across two orthogonal diameters perpendicular to the heated leg, 45 min after immersion. (b) Core temperatures of unheated and heated tumours, and rectal temperature, all measured in the same mouse.

relative weight of treated tumours was not significantly different from 1·0 up to 24 h after treatment.

### RESULTS

*Tumour temperature profile*

Fig. 1 shows temperatures measured in a typical tumour-bearing leg after immersion in water at 43·0°C. In Fig. 1a the temperatures across 2 orthogonal diameters were determined by drawing thermocouple probes through the leg. Leg diameters in the two "scans" were 10·3 mm (upper) and 10·7 mm (lower diagram). In this example, the temperature in the central part of the tumour was 1·2–1·6°C below the water temperature, even after 40min immersion. The temperature of a tumour in the contralateral unheated limb was also measured, and this, together with the mouse rectal temperature and temperature in the heated leg, is shown in Fig. 1(b). The temperature in the heated tumour reached its maximum after 15 min. The temperature in the unheated tumour did not exceed 36·7°C during 45 min immersion.

*Effect of heat alone on cell survival*

Fig. 2(a) shows SF, Relative Tumour-Cell Yield and $SF_{(tumour)}$ in B16 tumours excised 24 h after immersion in water at 43·0°C for 1 h. Fig. 2(b) gives similar data for Lewis lung tumours. Although the SF for cells recovered after heating is close to 100%, a substantial reduction was found in the $SF_{(tumour)}$. This indicates that cell death is rapid after hyperthermia. This can be taken into account when expressing the overall effect of the treatment on the tumour, as shown by $SF_{(tumour)}$. The median $SF_{(tumour)}$ was 0·21 (B16) and 0·125 (Lewis lung). For unheated tumours removed from mice whose opposite leg had been heated, the $SF_{(tumour)}$ was not significantly differ-

FIG. 2.—Cell survival in (a) B16 and (b) Lewis lung tumours 24 h after immersion in water at 43·0°C for 1 h.

ent from 1·0, showing that the heat treatment had only a direct local, not a systemic effect.

*Effect of combined heat and drugs on cell survival*

Cell survival was measured in B16 melanoma 24 h after treatment with graded doses of CCNU or melphalan, either alone or in combination with heating by immersion in water at 43·0°C for 1 h immediately after drug injection. The resulting dose–response curves are shown in Fig. 3. The upper curve in each diagram shows cell survival in tumours treated with the drug alone, administered without anaesthetic. For CCNU, this curve has a shoulder followed by an exponential decrease in cell survival with dose. For melphalan, the curve is apparently exponential. In hyperthermia experiments, concurrent cell survival assays were made on the heated tumour and the contralateral tumour not immersed in hot water. Both tumours were thus subjected to the same drug and anaesthetic dose, and their treatments differed only in the application of heat. The lower pair of curves in Fig. 3 show the cell survival in such unheated and heated B16 tumours after CCNU or melphalan.

Fig. 4 shows the results of similar experiments in which Lewis lung tumours were treated with CCNU or melphalan. All dose–response curves show an exponential decrease in cell survival with dose. In Fig. 4(b), data is shown for 2 different anaesthetics. The triangular symbols show the cell survival in tumours treated with melphalan with or without heat but using the anaesthetic Sagatal (sodium pentobarbitone) instead of Saffan. There is no significant difference in data between the 2 anaesthetics.

Table I gives the values of $D_{37}$, assessed by least-squares regression, for exponential parts of the survival curves in Figs 3 & 4. Table II summarizes the dose modifying factors (DMF: ratio of $D_{37}$ values) due to Saffan anaesthetic and the thermal enhancement ratios (TER) for the 2 tumours and 2 drugs. The DMF's for Saffan were calculated from the survival curves for the drug alone (upper curves) and the drug + anaesthetic (middle curves). TER's were calculated from the survival curves for the drug + anaesthetic (middle curves) and the drug + anaesthetic + heat (lower curves). TER was assessed as a DMF, *i.e.* a ratio of survival curve slopes. This method gives values, independent of cell survival, which are a measure of the enhancement of drug action on the tumours by heat. TER could also be calculated as a ratio of drug doses to give an isoeffect, and this method takes into account the direct cell killing by heat alone (the zero-drug dose intercept on the lower curves) and is an assessment of the total effect of the heat on the drug-treated tumours. TER values calculated by this method would therefore be larger. However, the value of TER determined for an isoeffect

FIG. 3.—Cell survival in B16 melanoma 24 h after treatment with (a) CCNU or (b) melphalan. Unheated tumours in conscious mice (□); unheated tumours (●) and heated tumours 43°C for 1 h (○) in Saffan-anaesthetized mice.

TABLE I.—*$D_{37}$ (mg/kg) for the dose–response curves of Figs.* 3 *&* 4

| | B16 melanoma | | Lewis lung carcinoma | |
|---|---|---|---|---|
| | CCNU | MEL | CCNU | MEL |
| Drug alone | 2·50 ± 0·50† | 2·05 ± 0·12 | 2·75 ± 0·07 | 3·20 ± 0·35 |
| Drug + Anaes. | 2·00 ± 0·08 | 1·15 ± 0·10 | 1·53 ± 0·07 | 1·19 ± 0·05 |
| Drug + Anaes. + Heat | 1·45 ± 0·16 | 0·74 ± 0·21 | 0·97 ± 0·09 | 0·49 ± 0·03 |

† Mean ± s.e.

TABLE II.—*Summary of DMF's for Saffan and TER's for B16 and Lewis lung tumours assayed* 24 *h after treatment. Heat* = 43 °*C waterbath,* 1 h

| | Saffan DMF | TER* |
|---|---|---|
| B16 | | |
| CCNU | 1·25 ± 0·25† | 1·38 ± 0·16 |
| Melphalan | 1·78 ± 0·19 | 1·55 ± 0·47 |
| Lewis lung | | |
| CCNU | 1·80 ± 0·09 | 1·58 ± 0·17 |
| Melphalan | 2·69 ± 0·32 | 2·43 ± 0·20 |

$* \frac{D_{37}\text{ (drug + anaes.)}}{D_{37}\text{ (drug + anaes. + heat)}}$ † Mean ± s.e.

is clearly dependent on the level of cell survival chosen and increases as the drug dose is decreased.

The most pronounced effect, both of the anaesthetic and the heat, was seen for melphalan treatment of the Lewis lung carcinoma. For the triple combination of melphalan anaesthetic and heat, the overall DMF was 6·5 relative to drug treatment in conscious animals ($D_{37}$ for melphalan alone/$D_{37}$ for Melphalan + anaesthetic + heat).

FIG. 4.—Cell survival in Lewis lung carcinoma 24 h after treatment with (a) CCNU or (b) melphalan. Unheated tumours in conscious mice (□); unheated tumours in mice anaesthetized by Saffan (●) or Sagatal (▲); heated tumours (43°C for 1 h) in mice anaesthetized by Saffan (○) or Sagatal (△).

*Effect of duration of heating and temperature on CCNU enhancement*

The increase in cell kill by CCNU, due to concurrent tumour heating, varied with both the duration of heating and the temperature of the water bath. This is shown in Fig. 5. In these experiments, mice bearing Lewis lung carcinoma were given a single i.p. dose of 7·5 mg/kg CCNU immediately after the start of heat treatment to one tumour-bearing limb. Although there is considerable scatter in cell survival after heat treatments in these experiments, it is clear that cell survival decreases with an increase in both heating time and water temperature. By contrast, in tumours growing in the opposite limbs, and therefore subject to the effects of the drug and the anaesthetic, but without direct heating, cell survival was about the same (1%) over the whole range of heating times and temperatures given to the other limb; and similar to the value without heat treatment (zero-time point, Fig. 5(a)). The observed effect of the anaesthetic on the tumour response to CCNU was therefore independent of any systemic effects due to local heating.

*Time course of cell killing by heat + CCNU*

The data in Fig. 6 indicate the cell survival after excising Lewis lung tumours at varying times after injection of a single dose of 7·5 mg/kg of CCNU. As in previous figures, the curves represent unheated unanaesthetized, anaesthetized but unheated, and anaesthetized and heated tumours (43·0°C water immersion, 1 h). The maximum cell-kill was found 3–4 h after treatment for all 3 experimental conditions, and was similar to the value measured at 24 h, suggesting no repair of CCNU damage during this time.

FIG. 5.—Cell survival in Lewis lung carcinoma 24 h after treatment with 7·5 mg/kg CCNU; with and without heating in water at 43·0°C for various times (a) or for 1 h in water at different temperatures (b). Unheated (●) and heated (○) tumours in Saffan-anaesthetized mice.

*Effect of drug scheduling relative to heat treatment*

The interaction of the heat and drug was investigated by varying the interval between a 1 h heat treatment (43·0°C water-immersion) and the time of CCNU administration. Cell survival 24 h after the drug injection in both heated and unheated Lewis lung tumours (from the same animals) is shown in Fig. 7. The response of the unheated tumours appears to remain unchanged when the interval between the anaesthetic and drug injection varied by up to 2 h. The response of the tumours to drug+heat, however, is schedule dependent; the maximum effect was seen when the drug was administered within 30 min before the start of heating.

DISCUSSION

We have found the cell yield in heated tumours to be consistently reduced to 5–20% of the untreated tumour value. Histological sections of tumours fixed 24 h after heating showed pycnotic and condensed cell nuclei and cell boundaries which appeared broken and ill-defined. In some heat-treated tumour sections there were small areas of apparently unaffected tissue. These observations are similar to those of Overgaard (1978). We conclude that the observed drop in tumour-cell yield is mainly due to rapid *in situ* degeneration of many cells after heat treatment. It is clear that this drop in tumour-cell yield must be taken into account when using an excision assay to measure cell survival, to obtain complete assessment of the killing effect of heat. The cell kill seen after heat alone in these experiments was manifest almost entirely as a reduction in cell yield (Fig. 2).

Our data on cell survival after drug treatment in the B16 and Lewis lung tumours agree well with those published previously (Stephens & Peacock, 1977; Peacock & Stephens, 1978). We have

FIG. 6.—Cell survival in Lewis lung carcinoma at various times after CCNU injection (7·5 mg/kg) combined with heat (43°C water-immersion, 1 h). Unheated tumours in conscious mice (△); unheated (●) and heated (○) tumours in Saffan-anaesthetized mice.

FIG. 7.—Cell survival in Lewis lung carcinoma 24 h after CCNU injection (7·5 mg/kg) and heat treatment (43°C water-immersion, 1 h) separated by up to 2 h. Unheated (●) and heated (○) tumours in Saffan-anaesthetized mice.

shown that heat (43·0°C water immersion, 1 h) increases tumour-cell killing by CCNU and melphalan, with TER's for slope ratios (relative to unheated tumours in the same animals) in the range 1·4–2·4. We have also seen a similar increased cytotoxicity in the contralateral unheated tumours as a result of using either Saffan or Sagatal anaesthetics. This enhanced cell killing appears to be the result of an interaction between the drug and anaesthetic, rather than a systemic effect of locally heating the contralateral leg, since equal cell killing was found, whether or not the opposite leg was heated (Fig. 5). These results extend those of Peacock & Stephens (1978) who found enhancement by Saffan of melphalan cell killing in the B16 melanoma. Clearly, hyperthermia studies involving chemotherapeutic drugs need to assess carefully the contribution of anaesthetics, if TER's are not to be over estimated. This can easily be done if the anaesthetic is given to animals bearing unheated tumours.

We cannot completely rule out the possibility that the thermal enhancement of cell killing seen in these experiments is an enhancement of the anaesthetic-drug interaction rather than the drug action alone. However, it is clear from Fig. 7 that while the effect of Saffan on CCNU cell killing remains constant if the anaesthetic is given up to 2 h before or after the drug, the thermal enhancement changes markedly with the relative timing of the heat and CCNU treatments. This does suggest that the heat directly affects the CCNU action. Furthermore, in another tumour system, Carcinoma NT, recent measurements of tumour-regrowth delay have demonstrated no significant effect of Sagatal on Melphalan-induced delay, suggesting *no* drug-anaesthetic interaction. However, a TER of 2·0 was found when melphalan was combined with local tumour heating for 1 h in water at 43·0°C (M. C. Joiner, unpublished data).

The cytotoxicity of CCNU did vary if the tumours were excised at different times up to $3\frac{1}{2}$ h after injection, though there was no change between $3\frac{1}{2}$ and 24 h. This appears to exclude inhibition of potentially lethal damage (PLD) repair as a mechanism for thermal enhancement of drug action. The results reported here are compatible with either an increase in drug delivery into the tumour cells or an increase in reaction rate between the drug and its target sites, as a result of heating. Hahn (1979) has reported thermal enhancement of cell killing by CCNU *in vitro* (HA1 Chinese hamster cells) and this suggests that heat interacts with this drug at a cellular rather than a physiological or tissue level.

Both CCNU and melphalan contain alkylating groups. In a study on the interaction of heat with the alkylating agent Thiotepa *in vitro*, Johnson & Pavelec (1973) suggested that, below 42°C, increased cell killing was compatible with a thermally-induced increase in the rate of alkylation. This then might also constitute a mechanism for the interaction of heat with CCNU or melphalan reported here.

Fig. 1 shows that in tumours immersed in hot water the temperature is not uniform, in agreement with Bleehen *et al.* (1977), Robinson *et al.* (1978) and Hill *et al.* (1980), all of whom have found intratumour temperature variations similar to those reported here. It is clear that temperature uniformity must be improved by the use of better heating methods, if scatter in results is to be reduced and, consequently, the mechanisms of interaction *in vivo* between heat and drugs clarified.

A criticism of temperature measurements is their invasive nature; *i.e.* a temperature probe might cause damage during insertion, so that readings were not representative of the true values in undisturbed tissues. Unfortunately, a technique for routine non-invasive thermometry is currently not available, and the problem is best approached by using very small probes to minimize tissue disturbance. In this study we have used probes with a diameter of 0·2 mm, which is very small compared to the diameter of the heated limbs (9·5–11·5 mm). Cetas & Connor (1978) have also reported that the use of temperature sensors mounted in hypodermic needles can lead to measurement errors due to thermal conduction along the metal sheath, and these errors can be important when using such probes for measurements in tissues at depths < 3 mm. We have therefore used only unsheathed thermocouple probes for the temperature measurements reported here.

In conclusion, we have demonstrated significant thermal enhancement of the cell-killing effects of CCNU and melphalan in two experimental tumours. It therefore appears that the use of mild local hyperthermia might be clinically beneficial in the management of tumours being treated with these drugs.

This work was supported by the Medical Research Council. We would like to thank Mr J. H. Peacock for assistance and discussion during the project and Dr J. Denekamp at the Gray Laboratory for helpful comments during the preparation of this paper.

### REFERENCES

Bleehen, N. M., Honess, D. J. & Morgan, J. E. (1977) Interaction of hyperthermia and the hypoxic cell sensitiser Ro-07-0582 on the EMT6 mouse tumour. *Br. J. Cancer*, **35**, 299.

Cetas, T. C. & Connor, W. G. (1978) Thermometry considerations in localized hyperthermia. *Med. Phys.* **5**, 79.

Courtenay, V. D. (1976) A soft agar assay for Lewis lung tumour and B16 melanoma taken directly from the mouse. *Br. J. Cancer*, **34**, 39.

Hahn, G. M. (1979) Potential for therapy of drugs and hyperthermia. *Cancer Res.*, **39**, 2264.

Hill, S. A., Denekamp, J. & Travis, E. L. (1980) Temperature non-uniformity in water-bath heated tumours. In *Proc. 1st Meeting Europ. Grp. Hyperthermia in Radiation Oncol.* Eds Arcangeli & Mauro. Milan: Masson Italia. p. 45.

JOHNSON, H. A. & PAVELEC, M. (1973) Thermal Enhancement of Thio-TEPA Cytotoxicity. *J. Natl Cancer Inst.*, **50**, 903.

MARMOR, J. B. (1979) Interactions of hyperthermia and chemotherapy in animals. *Cancer Res.*, **39**, 2269.

OVERGAARD, J. (1978) The effect of local hyperthermia alone, and in combination with radiation, on solid tumours. In *Cancer Therapy by Hyperthermia and Radiation*. Ed. Streffer *et al.* Munich: Urban & Schwarzenberg. p. 49.

PEACOCK, J. H. & STEPHENS, T. C. (1978) Influence of anaesthetics on tumour-cell kill and repopulation in B16 melanoma treated with Melphalan. *Br. J. Cancer*, **38**, 725.

ROBINSON, J. E., HARRISON, G. H., MCCREADY, W. A. & SAMARAS, G. M. (1978) Good thermal dosimetry is essential to good hyperthermia research. *Br. J. Radiol.*, **51**, 532.

STEEL, G. G. & ADAMS, K. (1975) Stem-cell survival and tumour control in the Lewis lung carcinoma. *Cancer Res.*, **35**, 1530.

STEPHENS, T. C., CURRIE, G. A. & PEACOCK, J. H. (1978) Repopulation of $\gamma$-irradiated Lewis lung carcinoma by malignant cells and host macrophage progenitors. *Br. J. Cancer*, **38**, 573.

STEPHENS, T. C. & PEACOCK, J. H. (1977) Tumour volume response, initial cell-kill and cellular repopulation in B16 melanoma treated with cyclophosphamide and 1-(2-chloroethyl)-3-cyclohexyl-1-nitrosourea. *Br. J. Cancer*, **36**, 313.

STEPHENS, T. C. & PEACOCK, J. H. (1978) Cell yield and cell survival following chemotherapy of the B16 melanoma. *Br. J. Cancer*, **38**, 591.